# Treatment of refractory hip pain with sodium hyaluronate (Hyalgan^©^) in a patient with the Marshall-Smith Syndrome: A case report

**DOI:** 10.1186/1749-799X-5-61

**Published:** 2010-08-23

**Authors:** Matthew Salter, Chandoo Kalmat, Henry Kroll, David Kim

**Affiliations:** 1 Henry Ford Hospital, Department of Anesthesiology, 2799 West Grand Boulevard, Detroit, MI, USA, 48202

## Abstract

The Marshall Smith Syndrome (MSS) is a rare congenital disorder, displaying a constellation of unique symptoms, including orofacial dysmorphisms, accelerated osseous maturation and dysplasias, mental retardation, and respiratory maladies. Few individuals with MSS survive past early childhood. In this case report, we describe a unique treatment for a 30 year-old patient with MSS who presented to our pain medicine clinic for management of pain secondary to uncontrolled bilateral hip dysplasias.

## Background

The Marshall-Smith Syndrome was first described in 1971 by Marshall et al as a rare congenital disorder, and to date there are fewer than 40 reported cases [1-3]. The etiology is unknown but is presumed to be due to a de novo dominant mutation. It is characterized by a constellation of features involving the neurologic and respiratory systems, and accelerated skeletal maturation leading to skeletal dysplasias. Patients have retarded intellectual development, small chins, glossoptosis, prominent eyes, protruding foreheads and are small in stature. They generally do not survive past early childhood mainly due to respiratory complications, such as aspiration pneumonia. However, if the respiratory conditions are managed aggressively, patients have been known to survive longer. To our knowledge, our patient is one of the oldest living patients with this rare disorder [3]. Given the natural history of the syndrome, one would anticipate that older subjects would suffer a great deal of pain as a result of the accelerated skeletal maturation. We report a unique treatment of incapacitating bilateral hip pain in a 30 year-old MSS patient with intra-articular hyaluronate (Hyalgan^©^).

## Case Report

L.W. is a 30 year-old woman with MSS. Her medical history was obtained from her parents, who accompanied her and upon whom she was totally dependent for care. The patient had speech and cognitive impairment that limited the ability to obtain a direct history. The parents described a history of worsening hip pain from progressive, bilateral hip dysplasias. Whereas previously their daughter could ambulate with assistance, she was now incapacitated by relentless pain. As best as they were able to determine, the pain radiated from her hips laterally, down her thighs and provoked regular paroxysms of screaming, crying and guarding.

Prior to our encounter, this pain had been managed by an orthopedic surgeon at an outside facility, who performed a series of nine ultrasound-guided intra-articular hip steroid injections, over a period of several years. The last one was performed a few months before presenting. None of the patient's previous records were available to us, but the parents relayed that their daughter's response to the injections had begun to wane with each repeat injection. The most recent recommendation from the orthopedist was to perform bilateral hip arthroplasties. The parents were hesitant to pursue this option, in light of their daughter's previous surgical experience, wherein she required an emergency tracheostomy after failed attempts at securing her airway under anesthesia. Because they were told that future surgeries would require an "awake" tracheostomy for airway protection during surgery, they decided to seek alternative, non-surgical treatments for their daughter's hip pain.

The rest of the review of systems was unremarkable. Notably, the parents denied any history of bleeding diathesis. Though previous diagnostic imaging was not available at the time of our initial consultation, they were reviewed at a later date, prior to treatment, and revealed dysplasia of both acetabula, and severe osteoarthritis and subluxation of both hip joints.

On physical examination, the patient was small in stature (4ft 2in, 65lbs) and had obvious craniofacial abnormalities. The neuromuscular exam was limited due to lack of patient cooperation. The greater trochanters were asymmetrical, with the right side about 2 cm superior compared to the left. Both were easily palpable and visibly appeared to be grossly out of socket. Though muscle tone was good with no flaccidity, the patient's inability to obey commands prevented us from assessing motor or sensory function. Reflex testing revealed patellar and Achilles hyporeflexia.

Based on the patient's history, physical examination, and radiographic findings, our impression was that the patient's symptoms arose directly from the articular surfaces of her hips, and possibly from the bilateral impingement of her lateral femoral cutaneous nerves, as a result of her inadequately developed acetabula and subluxed femurs. We suggested a series of three fluoroscopically guided intra-articular hip injections with sodium hyaluronate (Hyalgan^©^), administered weekly. In the event of an unsatisfactory result from the injections, we intended to perform bilateral lateral femoral cutaneous nerve blocks. The patient's parents elected to pursue injection of sodium hyaluronate, and scheduled an appointment for the procedure.

## Method

**Figure 1:**
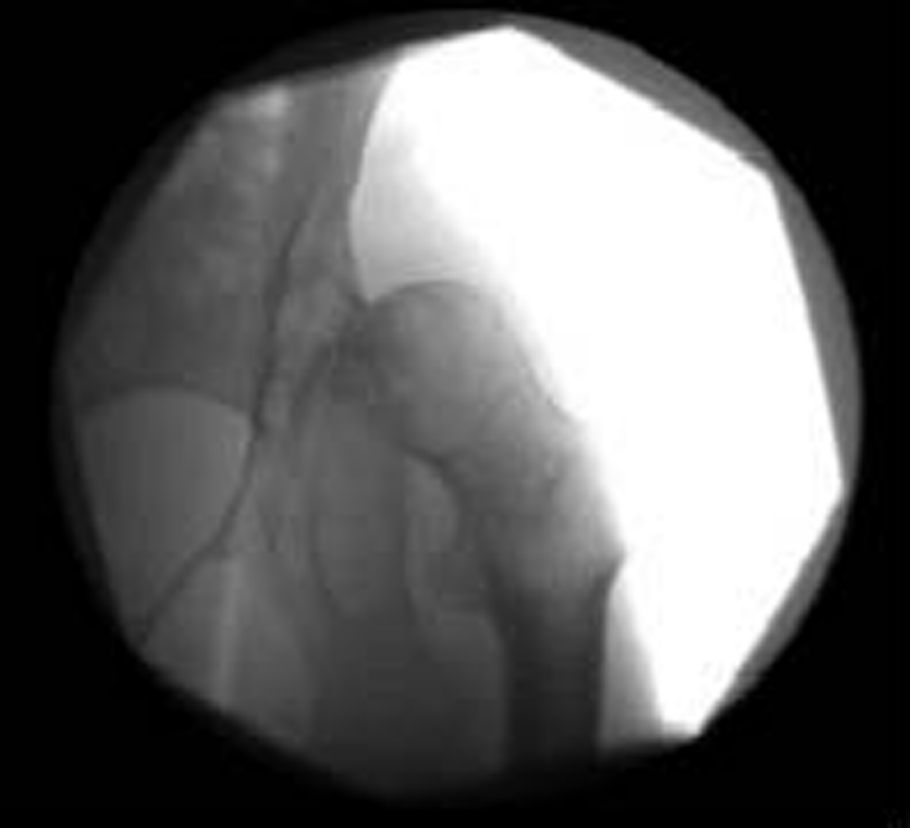
**A fluoroscopic image of the patient's right hip, taken prior to needle insertion for intra-articular hip injection**.

**Figure 2:**
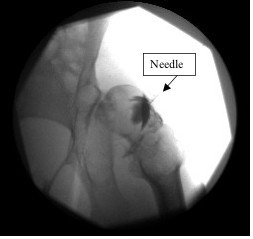
**A right hip fluoroscopic image with needle in place, showing spread of contrast within joint capsule**. Contrast spread is apparent from the base of the femoral neck and around joint.

After discussing the risks, benefits, and alternatives on the day of the procedure, informed consent was obtained from the patient's parents. Her parents were present during the entire procedure, to facilitate cooperation. After placement on the fluoroscopy table in the supine position, the region of the greater trochanters were cleansed with chlorhexidine (Cholraprep^©^) and draped fully. The fluoroscopic camera was positioned to visualize the right greater trochanter, femoral neck and acetabulum in the AP projection (Figure 1). Using a sterile marker, we marked the needle insertion site one centimeter cephalad to the greater trochanter. A 25-gauge, 1-1/2 inch needle was used to infiltrate the skin with 1% lidocaine. Subsequently, a 3-1/2 inch, 22-gauge spinal needle was advanced to the femoral neck and into the joint capsule at this level. Following this, after negative aspiration for blood, 1 milliliter (ml) of iopamidol-300 (Isovue-300^©^) dye was injected, verifying intra-articular spread of the dye (Figure 2). This was followed with an injection of 2 mL of 0.5% preservative-free bupivacaine and 2 ml (20 mg) of Hyalgan^© ^into the joint space. The same procedure was repeated on the left hip. The patient tolerated the procedure well without complications. We discharged the patient home after meeting discharge criteria. The patient returned to our clinic for a total of three injections of Hyalgan^©^, separated by one week.

## Discussion

**Figure 3:**
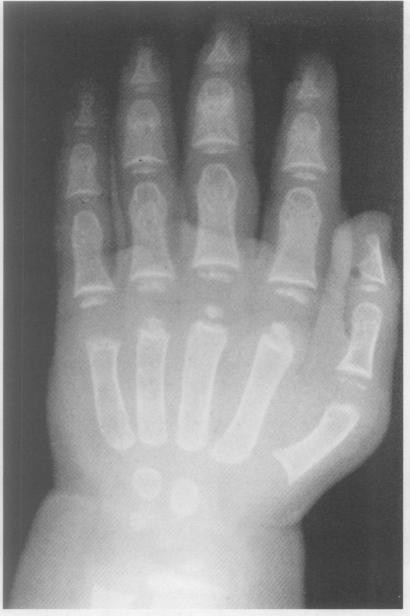
**A wrist/hand X-ray of a female with MSS, at chronological age of 18 weeks displaying wide phalanges & stippled epiphyses, showing bone age of 1.9 years **[9].

**Figure 4:**
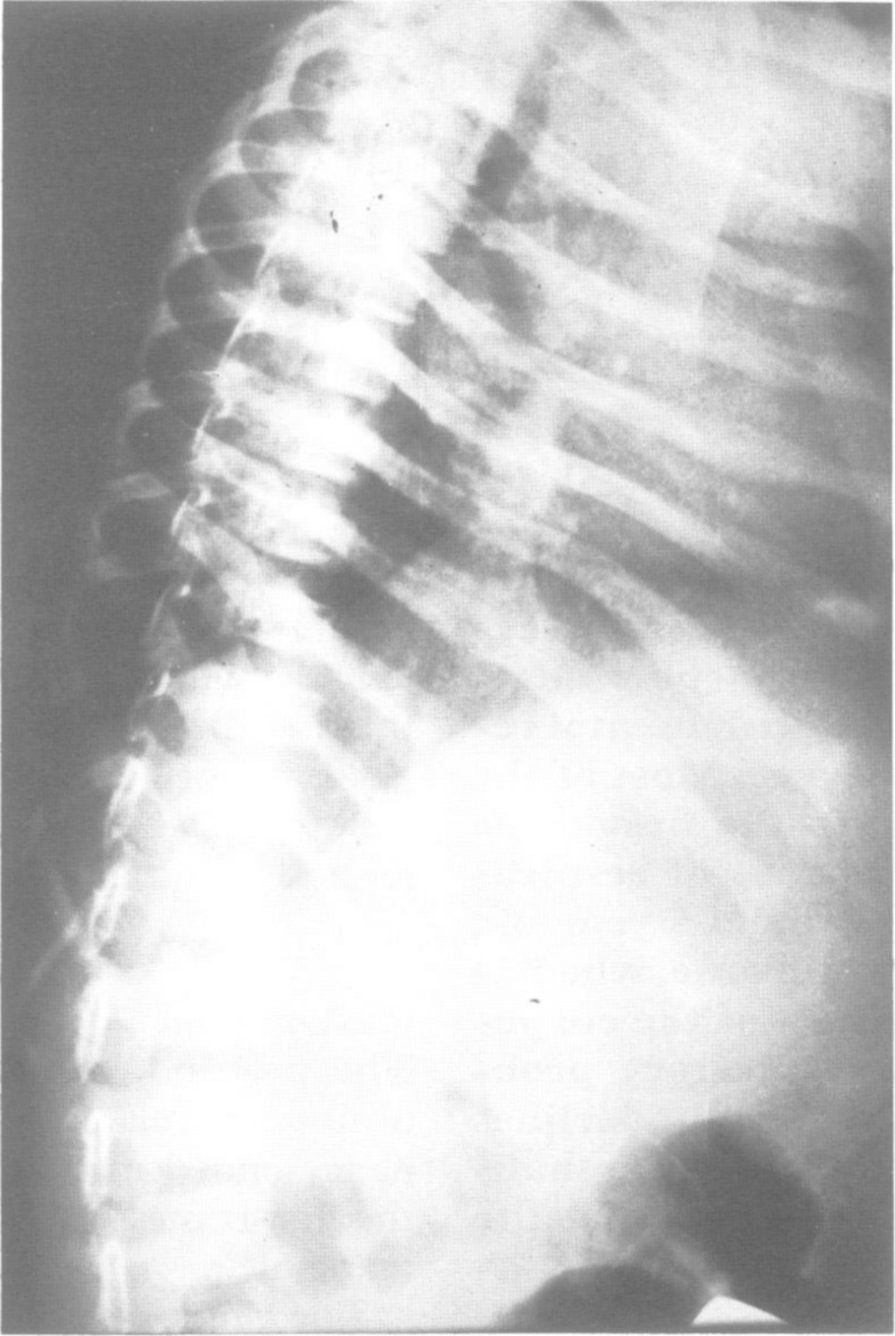
**An X-ray showing thinned ribs and dysmorphic lumbar vertebrae in a 4-week old male with MSS **[4].

**Figure 5:**
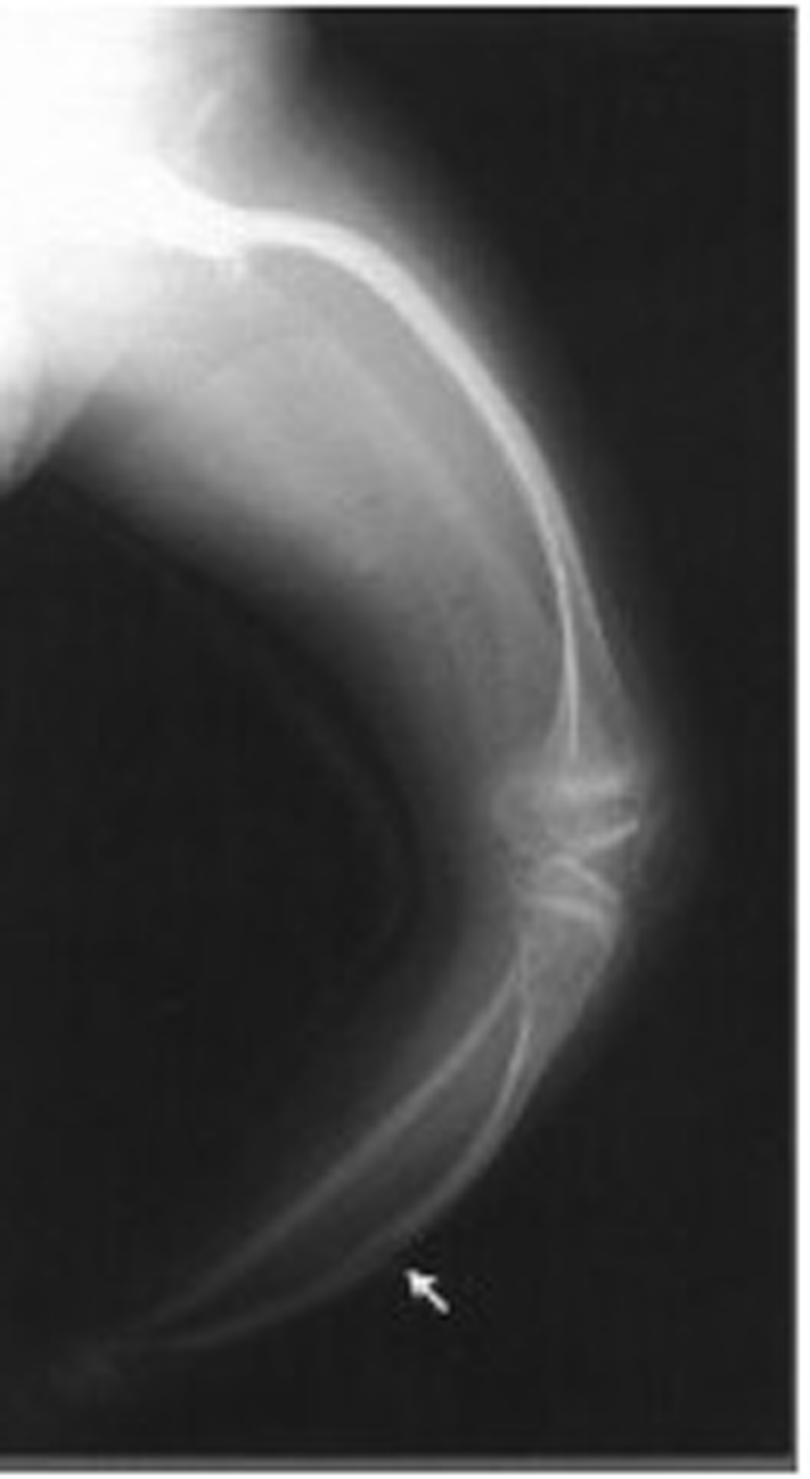
**Femur and Tibia X-ray of 7 year-old female with MSS demonstrating dysplastic hips with shallow, horizontal acetabula, post-traumatic bowing, Arrow indicates healing tibial fracture**. Diaphyses are gracile with thin cortices and obliterated medullae, in contrast with widened metaphyses and epiphyses, which are relatively spared [13].

**Figure 6:**
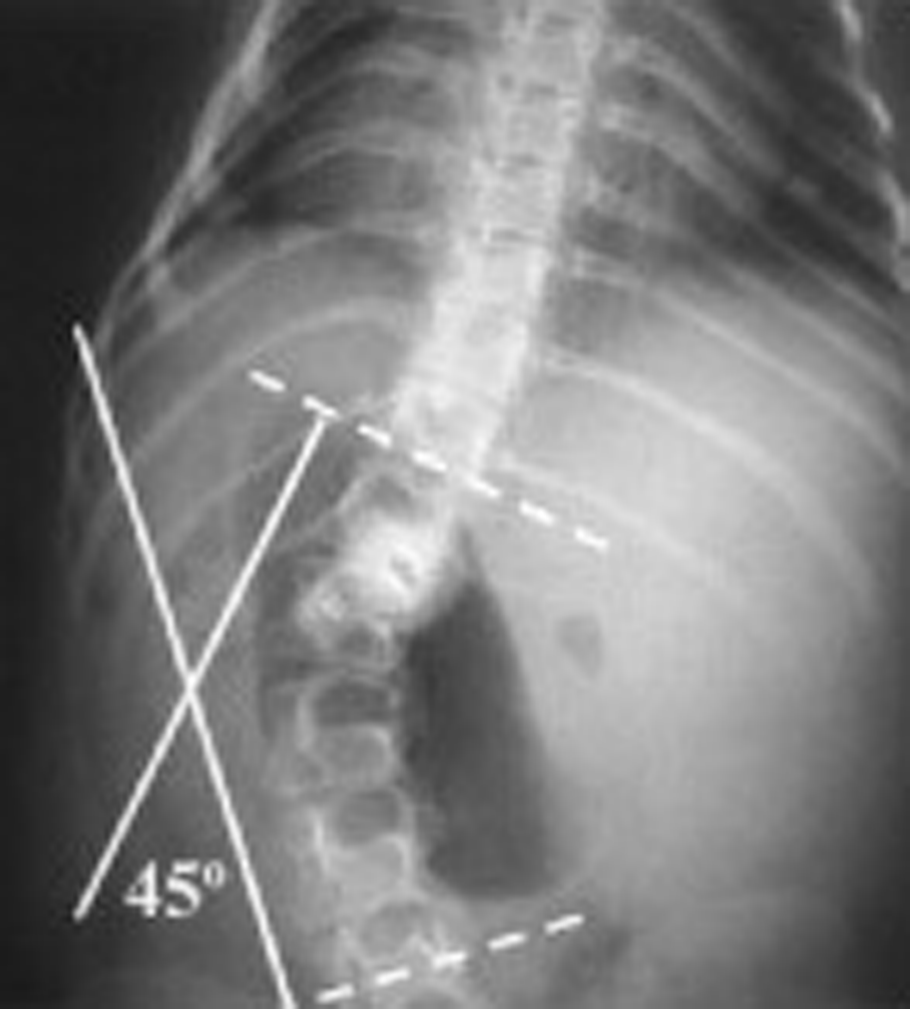
**X-ray of lumbar spine demonstrating an apex left 45 degree scoliosis in the same 7 year-old female with MSS **[13].

Marshall et al. first described this rare congenital disorder in 1971 as a sporadic entity of unknown etiology [4,5]. Patients classically have a normal karyotype and are born of nonconsanguineous parents [4]. Most significantly, they display skeletal maturation that is advanced for age, along with osteopenia and sclerosis, leading some to describe this kind of ossification as "disharmonic" [6-8]. Skeletal abnormalities like broadened phalanges with abnormal epiphyses, thinned or bowing long bones, and dysmorphic vertebrae leading to scoliosis, kyphosis, or cervical, thoracic, or lumbar instability are commonly present [[3-5,7-9], Figure 3, Figure 4, Figure 5, Figure 6]. Specific facial anomalies may include hypertrichosis, prominent eyes and forehead (frontal bossing), megalocornea, blue sclerae, a flat nasal bridge, micrognathia, and anteverted nostrils [5,10]. Neurologic derangements may consist of an absent corpus callosum, macrogyria, ventricular dilatation or hydrocephalus, periventricular leukomalacia, resulting in motor and mental retardation [5,10]. They might also display optic nerve hypoplasia [11]. It should be noted that not all patients afflicted with MSS display the same anatomic findings.

Most infants affected with MSS succumb to death early in life. This is usually due to pulmonary complications, such as aspiration and chronic lung obstruction leading to pulmonary infections, pulmonary hypertension, and right heart failure [2,4,5,10-13].

The concept of applying viscosupplementation (VS), using modified hyaluronic acid to form hyaluronans (HA) and their cross-linked derivatives, to the treatment of osteoarthritis arose in the mid 1970's, though these compounds had also been investigated for use in various ophthalmologic procedures [14-17]. As joints affected by osteoarthritis are depleted of their natural synovial fluid, which contains the glycosaminoglycan hyaluronic acid, it was postulated that injecting exogenous HA would increase the viscosity and elasticity to the joint, thereby improving joint function and relieving symptoms [18,19]. While research on, and FDA-approval of, VS is primarily for use in the treatment of knee osteoarthritis, it has also been used successfully in small trials for the treatment of arthritis of the temporomandibular, sacroiliac, hip, shoulder, foot, and ankle joints [17,20-23]. In 1997, Hyalgan^® ^(sodium hyaluronate), a high molecular-weight HA obtained from rooster's combs, was approved by the FDA [24,25]. Like other HA, it is only approved for osteoarthritis of the knee, and should be used with caution in patients with sensitivities to avian proteins, feathers, and egg products. It also carries a similar side-effect profile, including local inflammation, injection site pain and itching, anaphylaxis/anaphylactoid reactions, local ecchymosis, nausea/vomiting, diarrhea, anorexia, and headache [26]. While Hyalgan^© ^has a relatively short half-life of about twenty-four hours, the effect of the injections lasts for weeks, which suggests that it augments natural synovium production, mitigates nociception and inflammation, as well as increases the rheological properties (viscosity, elasticity, pseudoplasticity) of synovial fluid [27-29].

Our patient's survival to adulthood is somewhat anomalous, compared to the life expectancy of others afflicted with MSS. Therefore, we can only assume that if other carriers of the disease survived the historically perilous respiratory maladies of childhood, they too would suffer from the chronic pain of disharmonic skeletal development and ensuing arthralgias, as we observed in our patient, and as it has been noted in other carriers [13]. L.W. underwent a series of three intra-articular bilateral hip injections one week apart, and gradual improvement in symptoms over this period was noted. At the time of the first visit, the patient arrived in her wheelchair, and passive movement of her hips caused her great distress. Two months after the last injection, we reevaluated the patient in our clinic. She arrived for her appointment *walking*, semi-independently, with her parents on either side of her. They felt that the injections successfully decreased the frequency and intensity of her painful episodes, noting a marked improvement in her daily functioning.

## Conclusion

We describe a unique treatment alternative for a patient with Marshall Smith Syndrome and debilitating, painful bilateral hip dysplasias using intra-articular sodium hyaluronate injections. This management option should be considered in one's armamentarium, especially in the high-risk surgical population.

## Competing interests

The authors declare that they have no competing interests.

## Authors' contributions

MS and CK conceived the project and conducted the primary literature review and manuscript composition. HK and DK contributed additional data to the literature review and manuscript. All authors read and approved the final manuscript.

## Consent

Written informed consent was obtained from the parent/guardian of the patient for publication of this case report and accompanying images. A copy of the written consent is available for review by the Editor-in-Chief of this journal.
